# Xanthine oxidoreductase gene polymorphisms are associated with high risk of sepsis and organ failure

**DOI:** 10.1186/s12931-023-02481-8

**Published:** 2023-07-06

**Authors:** Li Gao, Nicholas Rafaels, Tanda M. Dudenkov, Mahendra Damarla, Rachel Damico, James P. Maloney, Marc Moss, Greg S. Martin, Jonathan Sevransky, Carl Shanholtz, Dan L. Herr, Joe G.N. Garcia, Tamara Hernandez-Beeftink, Jesús Villar, Carlos Flores, Terri H. Beaty, Roy Brower, Paul M. Hassoun, Kathleen C. Barnes

**Affiliations:** 1 Department of Medicine, The Johns Hopkins University School of Medicine, Baltimore, MD USA; 2 Division of Biomedical Informatics & Personalized Medicine, University of Colorado School of Medicine, Aurora, CO USA; 3 Department of Epidemiology, Bloomberg School of Public Health, The Johns Hopkins University, Baltimore, MD USA; 4 Division of Pulmonary Sciences and Critical Care Medicine, University of Colorado School of Medicine, Aurora, CO USA; 5 Department of Medicine, Emory University School of Medicine, Atlanta, GA USA; 6 University of Maryland School of Medicine, Baltimore, MD USA; 7 University of Arizona College of Medicine, Tucson, AZ USA; 8 Research Unit, Hospital Universitario Ntra. Sra. de Candelaria, Santa Cruz de Tenerife, Spain; 9 Research Unit, Hospital Universitario Dr. Negrin, Las Palmas de Gran Canaria, Spain; 10 CIBER de Enfermedades Respiratorias (CIBERES), Instituto de Salud Carlos III, Madrid, Spain; 11 Li Ka Shing Knowledge Institiute at St. Michael’s Hospital, Toronto, Canada; 12 Genomics Division, Instituto Tecnológico y de Energías Renovables, Santa Cruz de Tenerife, Spain; 13 Facultad de Ciencias de la Salud, Universidad Fernando Pessoa Canarias, Las Palmas de Gran Canaria, Spain; 14 The Johns Hopkins Asthma & Allergy Center, 5501 Hopkins Bayview Circle, Room 3B.65B, Baltimore, MD 21224 USA; 15 University of Colorado Anschutz Medical Campus, 13001 E. 17th Place, Room 5330A, Aurora, CO 80045 USA

**Keywords:** Sepsis, Acute respiratory distress syndrome, Xanthine oxidoreductase, Single nucleotide polymorphism, Haplotype, Biomarker

## Abstract

**Background:**

Sepsis and associated organ failures confer substantial morbidity and mortality. Xanthine oxidoreductase (XOR) is implicated in the development of tissue oxidative damage in a wide variety of respiratory and cardiovascular disorders including sepsis and sepsis-associated acute respiratory distress syndrome (ARDS). We examined whether single nucleotide polymorphisms (SNPs) in the *XDH* gene (encoding XOR) might influence susceptibility to and outcome in patients with sepsis.

**Methods:**

We genotyped 28 tag SNPs in *XDH* gene in the CELEG cohort, including 621 European American (EA) and 353 African American (AA) sepsis patients. Serum XOR activity was measured in a subset of CELEG subjects. Additionally, we assessed the functional effects of *XDH* variants utilizing empirical data from different integrated software tools and datasets.

**Results:**

Among AA patients, six intronic variants (rs206805, rs513311, rs185925, rs561525, rs2163059, rs13387204), in a region enriched with regulatory elements, were associated with risk of sepsis (*P* < 0.008–0.049). Two out of six SNPs (rs561525 and rs2163059) were associated with risk of sepsis-associated ARDS in an independent validation cohort (GEN-SEP) of 590 sepsis patients of European descent. Two common SNPs (rs1884725 and rs4952085) in tight linkage disequilibrium (LD) provided strong evidence for association with increased levels of serum creatinine (*P*_*adjusted*_<0.0005 and 0.0006, respectively), suggesting a role in increased risk of renal dysfunction. In contrast, among EA ARDS patients, the missense variant rs17011368 (I703V) was associated with enhanced mortality at 60-days (*P* < 0.038). We found higher serum XOR activity in 143 sepsis patients (54.5 ± 57.1 mU/mL) compared to 31 controls (20.9 ± 12.4 mU/mL, *P* = 1.96 × 10^− 13^). XOR activity was associated with the lead variant rs185925 among AA sepsis patients with ARDS (*P* < 0.005 and *P*_*adjusted*_*<*0.01). Multifaceted functions of prioritized *XDH* variants, as suggested by various functional annotation tools, support their potential causality in sepsis.

**Conclusions:**

Our findings suggest that XOR is a novel combined genetic and biochemical marker for risk and outcome in patients with sepsis and ARDS.

**Supplementary Information:**

The online version contains supplementary material available at 10.1186/s12931-023-02481-8.

## Background

Sepsis is an organ dysfunction syndrome in response to intravascular or extra-vascular microbial agents. Sepsis is a serious public health problem in the United States and globally [1–3], with an associated mortality above 30% [4, 5]. Recent studies have highlighted risk factors and ethnic differences [6–9], with African Americans (AA) having higher risk of death compared to European American (EA) patients, suggesting potential genetic influences for risk and outcome of sepsis. The degree and number of organ dysfunctions have an additive effect on mortality [6, 10]. Thus, studies identifying novel genetic and biochemical markers may provide insights into the pathogenesis of sepsis, allowing personalized therapies.

Oxidant injury related to excess formation of reactive oxygen species (ROS), either generated by xanthine oxidoreductase (XOR) or other oxidant-producing enzymes, might play a role in the pathogenesis of sepsis-associated acute respiratory distress syndrome (ARDS) [11–14]. Oxidative stress activates the redox-sensitive transcription factors (e.g., NF-қB and AP-1), resulting in a large output of proinflammatory cytokines and chemokines, which further aggravate inflammation and oxidative stress [15]. In experimental models of sepsis and ARDS, endotoxin exposure produces a significant increase in lung XOR mRNA and enzymatic activity while causing lung injury, which can be successfully prevented by pharmacologic inhibition of XOR [16]. Increased hypoxanthine and XOR activities have been observed in ARDS patients compared with normal controls or critically ill patients with other diseases [14]. Furthermore, plasma hypoxanthine (XOR substrate) levels are highest in patients who die from ARDS, suggesting oxidative damage (and presumably XOR) as implicated in outcome [17]. However, there has been little progress in anti-oxidative strategies [18], and specifically XOR-targeted therapy, in clinical trials perhaps due to a lack of accurate tools for identifying high-risk patients who might benefit from such interventions.

Genes encoding regulators of the oxidant-mediated response are implicated in the risk for sepsis [19]. After reporting an association between single nucleotide polymorphisms (SNPs) in the gene encoding the transcription factor *Nrf2* (NF-E2 related factor 2) and regulation of oxidant-mediated response in ARDS [20], two redox genes were subsequently reported in association with ARDS (manganese superoxide dismutase, MnSOD; and superoxide dismutase 2 SOD2) [21, 22]. Genetic variation is also associated with outcome disparity in critical illness, including sepsis [23]. We hypothesized that *XDH* SNPs may represent risk factors for the development of sepsis and associated organ failure by affecting tissue XOR activity, thus serving as biomarkers of disease severity and outcome.

## Methods

### Study subjects

This is a genetic study on sepsis and ARDS including 974 patients (EA = 621, AA = 353; Table 1) from six clinical sites participating in the Consortium to Evaluate Lung Edema Genetics (CELEG), approved by human subject research committees at Johns Hopkins University and other enrollment sites [24–26]. The CELEG study enrolled patients with sepsis or septic shock, a subset of whom also had sepsis-associated ARDS (Supplementary Methods, Additional File 1). A total of 621 EA septic patients with (n = 297) or without (n = 324) ARDS, and an independent AA dataset of 353 sepsis patients (158 with ARDS, 195 without ARDS), were enrolled in this study. A group of controls (EA = 302, AA = 406) were also enrolled and included in genotyping to estimate minor allele frequencies (MAF), linkage disequilibrium (LD) structures and individual ancestries.

**Table 1:** Demographics and clinical characteristics of sepsis patients with and without ARDS in CELEG population

	Total			Sepsis (w/o ARDS)			Sepsis (w/ ARDS)		
	European American	African American	*P*	European American	African American	*P*	European American	African American	*P*
**N**	621	353		324	195		297	158	
**Sex (M/F)**	352/269	176/177	0.05	175/149	89/106	0.07	177/120	87/71	0.37
**Age (yrs)**	60.0 ± 16.7	54.8 ± 17.3	*< 0.0001*	62.6 ± 15.8	58.9 ± 16.2	0.01	57.3 ± 17.2	49.7 ± 17.2	*< 0.0001*
**APACHE II**	25.7 ± 8.0	25.3 ± 8.1	0.38	23.9 ± 8.1	23.8 ± 7.4	0.87	27.8 ± 7.5	27.1 ± 8.6	0.42
**Mortality (%)**	32.53	36.54	0.21	26.23	26.15	1.0	39.39	49.37	0.05
**Insult (%)** Lung Blood Abdomen UTI Other	302 (49.6) 70 (11.5) 88 (14.4) 72 (11.8) 77 (12.6)	150 (44) 60 (17.6) 38 (11.1) 39 (11.4) 54 (15.8)	0.03	105 (33.1) 42 (13.2) 55 (17.4) 61 (19.2) 54 (17)	62 (32.8) 36 (19) 23 (12.2) 29 (15.3) 39 (20.6)	0.16	197 (67.5) 28 (9.6) 33 (11.3) 11 (3.8) 23 (7.9)	88 (57.9) 24 (15.8) 15 (9.9) 10 (6.6) 15 (9.9)	0.13
**Comorbidities (%)**									
HIV	4.52	17.85	*< 0.0001*	3.10	15.90	*< 0.0001*	6.06	20.25	*< 0.0001*
AIDS	2.58	11.90	*< 0.0001*	2.17	8.72	*< 0.0001*	3.03	15.82	*< 0.0001*
RF	45.48	44.76	0.84	43.65	44.62	0.85	47.47	44.94	0.62
CLD	12.90	4.82	*< 0.0001*	10.22	4.10	*0.01*	15.82	5.70	*0.002*
Diabetes	26.45	35.41	*0.004*	30.65	43.08	*0.01*	21.89	25.95	0.35
Alcoholism	16.13	18.98	0.29	11.15	16.41	0.11	21.55	22.15	0.91
COPD	21.29	10.76	*< 0.0001*	21.67	10.77	*0.002*	20.88	10.76	*0.006*
Cancer	19.68	12.75	*0.01*	23.22	10.77	*< 0.0001*	15.82	15.19	0.89
CHF	11.29	11.08	1.0	11.76	11.79	1.0	10.77	10.19	1.0
Anemia	18.55	17.00	0.60	19.50	21.03	0.73	17.51	12.03	0.14

Total cohort = 974 subjects, definition of abbreviations: APACHE II = Acute Physiology and Chronic Health Evaluation; CLD = Chronic Liver Disease; RF = Renal Failure; UTI = Urinary Tract Infection; CNS = Central nervous system; Age and APACHE II data expressed as mean ± SD; Mortality is measured up to 60 days

Two group comparisons were carried out using either a two-tailed Student’s t-test for age and APACHE II score or Fisher’s exact test for categorical variables (sex and survival). Chi-square test was applied for clinical variable insult. *P* values under the Total, Sepsis and ARDS heading refer to differences between races. Significance level was set at *P* ≤ 0.05

### Genotyping

**Fig. 1:**
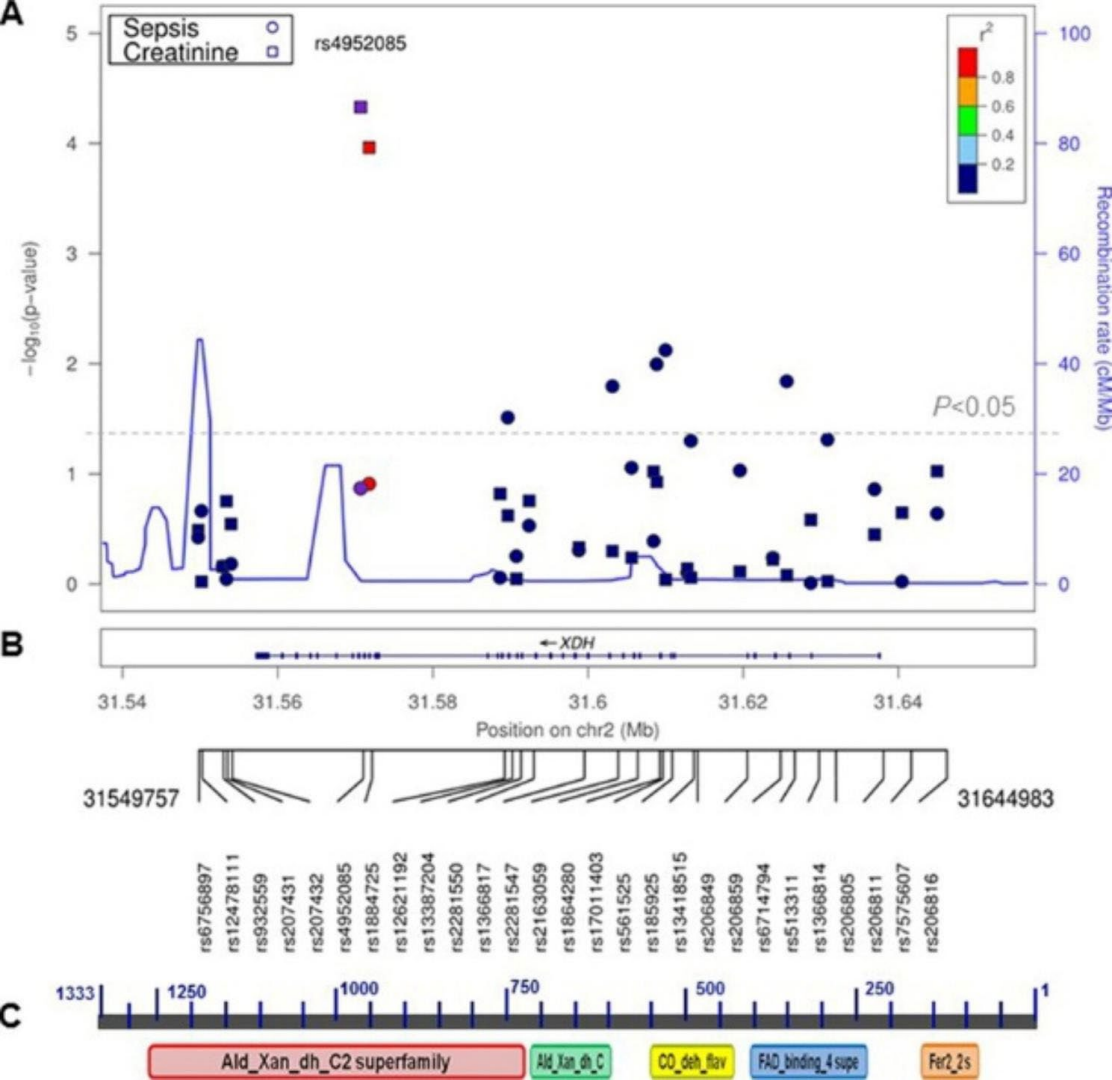
Summary distributions of -log_10_ (*P* value) for association between *XDH* SNPs and risk, renal dysfunction among African American patients with sepsis. ***Top panel***: A locus-zoom plot of the top loci from single marker association analysis for risk of sepsis and renal dysfunction. The Y axis indicates the negative log of the *P* value with recombination rates estimated from the 1000 Genomes Project CEU data, and the X axis indicates the genomic position for SNPs genotyped in *XDH*, 3’ to 5’ direction (on reverse strand). The circle and square in black represent signals for risk of sepsis and serum creatinine levels, respectively; the two SNPs associated with creatinine concentration are distinguished in purple. SNP rs4952085 is used as index SNP, and the r^2^ values of remaining SNPs are indicated by color. The grey horizontal line represents the significance level of *P* = 0.05. ***Middle panel*** illustrates the *XDH* gene structure on chromosome 2 and relative positions of 28 SNPs in the gene. ***Bottom panel*** shows conserved domains (Fer2_2 S, FAD_binding_4 supe, CO_deh_flav, Ald_Xan_ dh_c and Ald_Xan_dh_C2 superfamily) on human xanthine dehydrogenase/oxidase (NP_000370)

DNA was extracted using standard protocols. Genotyping was performed using the GoldenGate platform (Illumina Inc., San Diego, CA), with an average completion rate of 98% and no discordances upon repeat genotyping of a random 2% of samples. Twenty-eight SNPs within the *XDH* gene and ~ 8 kb up and downstream were selected and genotyped in cases and controls (Fig. 1 & Supplementary Tables 1, Additional File 1). Of these markers, 27 are Illumina tag SNPs selected in accordance with the Illumina Assay Design Tool which included: (1) SNP design score (≥ 0.6); (2) MAF ≥ 0.05; and (3) Inter-SNP distance (≥ 60 bp). We also included the only coding non-synonymous SNP rs17011368 (Ile703Val). An additional 40 ancestry informative markers (AIMs) were genotyped to evaluate genetic stratification of AA group [25].

### Serum XOR activity in patients

Blood samples for serum isolation were collected when diagnosis criteria were met at two of the six clinical sites for a total of 143 patients (AA = 53, EA = 90). Additional AA controls (N = 31) were collected at the John Hopkins site only. Serum isolation process was completed within 4 h, and then tubes were transferred to -80^o^C for storage. XOR activity was measured and analyzed in duplicate with an initial 2-fold dilution using the Amplex Red reagent-based assay (Molecular Probes, Eugene, OR). After 60-min incubation at 37^o^C, absorbance was read on a microplate reader (BioRad, model 680) at 570 nm. XOR activity in serum was log transformed, and the difference between groups was analyzed using Fisher’s exact test.

### Statistical analysis

Descriptive statistics for demographics and clinical characteristics were calculated using STATA 9.0 (StataCorp. College Station, TX). Departures from Hardy-Weinberg equilibrium (HWE) proportions at each SNP were tested in cases and controls separately using default parameters in Haploview v3.2 [27]. Haploview was also used to examine patterns of LD. Individual ancestries in AA subjects were estimated using STRUCTURE 2.1 [28]. To test for association between genetic markers in *XDH* and disease risk among septic patients, we used multivariate logistic regression analysis, including age, sex, and genetic ancestry as covariates. Associations between *XDH* markers and levels of serum creatinine (assessing renal dysfunction) or XOR activity were tested using linear regression. Age was recorded as a categorical variable with three categories: 40 years or less, 41 to 59 years, and 60 years or greater. In addition, we used logistic regression models to test for marker association with mortality (treated as dichotomous trait) assuming an additive risk model. Sliding-window haplotype analyses were performed using an algorithm implemented in PLINK [29]. Survival curves in genotype groups were computed in ARDS patients to examine time-to-death by using the Kaplan-Meier estimates and evaluated by the log-rank test.

### Genetic association testing in the GEN-SEP cohort

Sepsis was defined according to the Third International Consensus Definitions for Sepsis [30]. Patients were admitted into a network of ICUs in Spain (GEN-SEP). This cohort was previously used to perform a sepsis-associated ARDS GWAS on 590 unrelated patients (274 sepsis-associated ARDS cases and 316 controls with sepsis only) [31]. SNPs data were obtained for 587,352 sites using the Axiom Genome-Wide Human CEU 1 Array (Affymetrix, Santa Clara, CA, USA). SNPs were imputed based on the HRC release 1.1.0, and variants with low allele frequency (MAF < 1%) or with a low imputation quality (Rsq < 0.3) were excluded from the analysis. The methods for testing genetic associations in this study, for ARDS or sepsis, have been described elsewhere [31].

### Functional annotation of genetic variants

We applied several *in-silico* tools to predict potential regulatory elements in the genomic region encompassing *XDH*, including epigenetic modifications (RegulomeDB [32] and SiNoPsis [33]) and tissue-specific local eQTLs (Genotype-Tissue Expression Project [GTEx] datasets). An additional tool (Open Targets Genetics [34]) was used to predict the likelihood of deleteriousness of each variant.

## Results

### Patient characteristics

Main clinical characteristics of CELEG subjects are listed in Table 1. As reported previously [25], age varied between racial groups within each diagnosis. Severity of illness and mortality were higher among septic ARDS as compared to sepsis-only patients in both ethnic groups (*P* < 0.0001). AA patients with sepsis-associated ARDS had the highest 60-day mortality (49.4%). Additionally, AA septic patients had higher serum creatinine concentration than EAs (3.0 ± 2.5 vs. 2.2 ± 1.9 mg/dL, *P* < 0.0001).

### *XDH* polymorphisms confer high risk for sepsis among african american patients

Six intronic variants (rs206805, rs513311, rs185925, rs561525, rs2163059, rs13387204) showed an association with sepsis in AAs compared to controls (*P* < 0.008–0.049) [Table 2; Fig. 1 (panels A and B)]. This association was absent among EA patients. Three out of six variants (rs185925, rs561525, rs2163059), providing most compelling evidence for association, were centered on exons 8 to 12, a region of high LD among AAs compared to EAs. Of note, the three top SNPs were clustered in a region enriched with regulatory features. These included DNAase I hypersensitivity clusters, transcription factors binding sites and enhancer histone H3K27Ac marks (which are often found near active regulatory elements), according to the evaluation of regulatory regions using the Encyclopedia of DNA Elements (ENCODE) regulatory tracks (Supplementary Fig. 2, Additional File 1) [35]. Our findings suggest potential functional consequences for these SNPs.

**Table 2:** Association between genetic variations in *XDH* gene and risk of development of sepsis among 353 African American sepsis patients (with and without ARDS) and 406 control subjects

Marker	Chr.2 Position (hg19)	Function	Alleles	MAF (case/control)	OR (95% CI)	*P*crude	Padj*
rs206805	31,630,867	intron 1	A/G	0.214/0.274	0.761 (0.579–0.999)	0.014	0.049
rs513311	31,625,610	intron 3	A/C	0.076/0.125	0.609 (0.409–0.906)	0.004	0.014
rs185925	31,609,993	intron 8	C/T	0.297/0.23	1.464 (1.107–1.936)	0.004	0.005
rs561525	31,608,855	intron 9	C/T	0.057/0.099	0.552 (0.351–0.869)	0.005	0.010
rs2163059	31,603,134	intron 12	A/G	0.479/0.412	1.35 (1.057–1.724)	0.010	0.016
rs13387204	31,589,654	intron (boundary)	C/T	0.297/0.354	0.759 (0.591–0.975)	0.027	0.031

*P values adjusted for age, gender and African ancestry

Major alleles for each SNP tested in the African Americans were considered as the reference alleles

### *XDH* polymorphisms are associated with renal dysfunction among african american patients

**Fig. 2:**
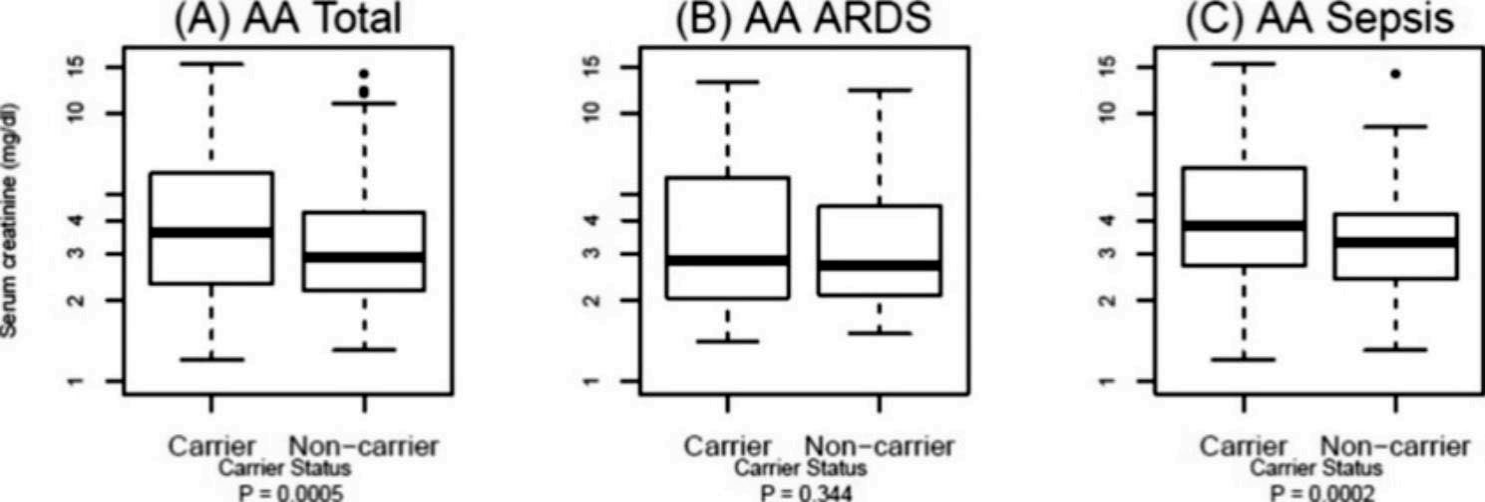
Comparison of serum creatinine levels (normal range: 0.5–1.2 mg/dL). Increased levels of serum creatinine were observed for carriers compared to non-carriers among African American sepsis patients stratified by either rs4952085 or rs1884725 genotypes (***panel A***). African American sepsis-only patients who were carriers had the highest serum creatinine levels (4.07 ± 1.78, n = 71) compared to non-carriers (3.24 ± 1.58, n = 99) (*P* < 0.0002, ***panel C***). No difference was observed in sepsis patients with ARDS (***panel B***). Mean and SD values are presented

We found two markers (a coding-synonymous SNP rs1884725 in exon 27 and another SNP rs4952085 in intron 27) in tight LD (r^2^ = 0.983) associated with increased serum creatinine concentration (regression coefficient beta = 0.504 and 0.493, respectively) in 353 AA sepsis patients (*P*_*adjusted*_ <0.0005 and 0.0006, respectively) (Table 3). With each additional copy of the *risk* alleles (‘A’ for rs1884725 or ‘G’ for rs4952085), the average increase in serum creatinine concentration was 0.5 mg/dL (normal range: 0.5–1.2 mg/dL). Table 4; Fig. 2 report serum creatinine concentration among AA patients (n = 310) stratified by rs1884725 or rs4952085 genotypes: this model predicted increased serum creatinine in patients carrying the *risk* genotypes (AA and GA genotypes for rs1884725, or GG and AG genotypes for rs4952085; 3.5 ± 2.9, n = 125) compared to non-carriers (2.6 ± 2.2, n = 185; *P <* 0.0005) (Fig. 2, panel A). Among them, sepsis-only AA patients who were carriers had the highest creatinine concentration (3.8 ± 2.8, n = 71) compared to the non-carriers (2.6 ± 1.9, n = 99; *P <* 0.0002, Fig. 2, panel C). In contrast, no difference was observed between carriers and non-carriers for creatinine concentration among sepsis patients with ARDS (Fig. 2, panel B).

**Table 3:** Association with renal dysfunction (serum creatinine level) among 353 African American sepsis patients

Marker	Chr. 2 Position (hg19)	Function	Alleles	MAF	Beta* (95% CI)	*P*crude	Padj**
rs4952085	31,570,689	intron 27	A/G	0.226	0.493 (0.215–0.771)	0.015	0.0006
rs1884725	31,571,786	coding-synonymous (exon 27)	G/A	0.225	0.504 (0.224–0.784)	0.015	0.0005

*Beta: regression coefficient, with each additional copy of the minor *risk* alleles (the ‘G’ or ‘A’ allele for rs4952085 or rs1884725, respectively), the average increase in serum creatinine concentration was 0.5 mg/dL (normal range: 0.5–1.2 mg/dL)

^**^Linear regression generated *P* values adjusted for demographic characteristics (age, sex and African ancestry) and APACHE II scores

**Table 4:** The serum creatinine level (mg/dL) among sepsis patients stratified by carrier status of either rs1884725 or rs4952085 in XDH*

	African Americans			European Americans		
	Total	Carriers	Non-carriers	Total	Carriers	Non-carriers
**All sepsis**	3.00 (2.5); 310	3.54 (2.9); 125	2.64 (2.2); 185	2.18 (1.8); 593	2.16 (1.7); 342	2.19 (1.9); 251
**Sepsis w/o ARDS**	3.13 (2.4); 176	3.78 (2.8); 77	2.63 (1.9); 99	2.26 (2.0); 310	2.31 (2.0); 122	2.23 (2.1); 129
**Sepsis w/ ARDS**	2.83 (2.6); 134	3.16 (2.9); 48	2.65 (2.4); 86	2.08 (1.6); 283	2.02 (1.5) 188	2.14 (1.8); 154

*Data was displayed as mean (SD); n

### Increased serum XOR activities in sepsis patients and association with variants

**Fig. 3:**
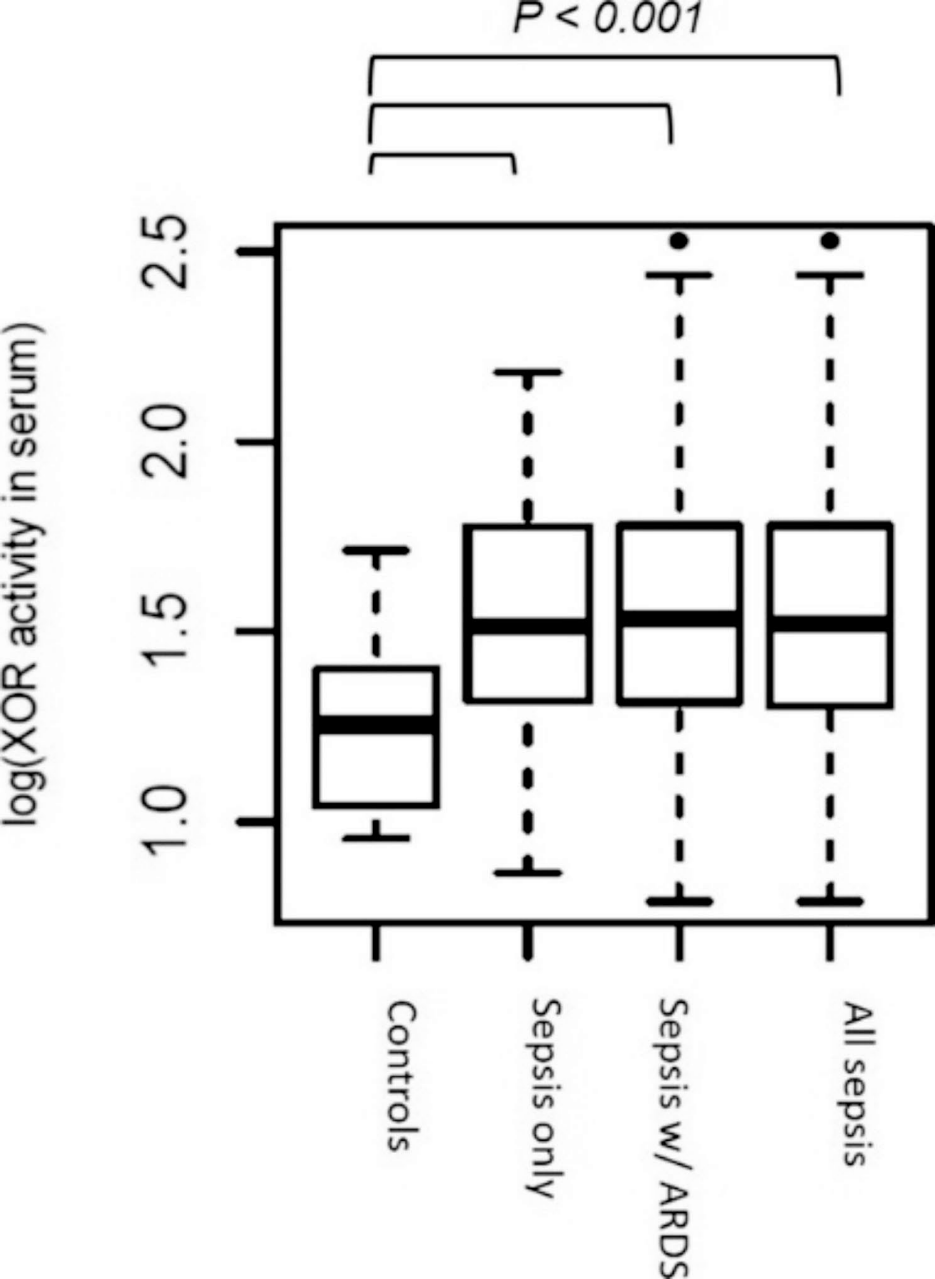
Serum XOR activity. Increased serum XOR activity was observed in the combined group of all sepsis patients (54.49 ± 57.09 mU/mL; n = 143), sepsis-only patients (43.76 ± 27.9 mU/mL; n = 68) or sepsis patients with ARDS (73.35 ± 64.91 mU/mL; n = 75) as compared to controls (20.85 ± 12.43 mU/mL; n = 31) (*P* = 1.96 × 10^− 13^, *P* < 0.0002 and *P* < 0.0001, respectively)

In a subset of patients from the CELEG cohort, we evaluated the correlation between XOR activity and disease status. Increased serum XOR activity was observed (Fig. 3) in the combined group of all patients (54.5 ± 57.1 mU/mL; or log-transformed: 1.6 ± 0.4; n = 143), sepsis-only patients (43.8 ± 27.9 mU/mL; or log-transformed: 1.5 ± 0.3; n = 68) or sepsis with ARDS (73.4 ± 64.9 mU/mL; or 1.6 ± 0.4; n = 75) as compared to the controls (20.9 ± 12.4 mU/mL, or 1.3 ± 0.2; n = 31) (*P* = 1.96 × 10^− 13^, *P* < 0.0002 and *P* < 0.0001, respectively). Of note, these patterns remained significant when we stratified patients by their carrier status of the lead variant rs185925. Most importantly, sepsis patients with ARDS who were homozygous carriers of the alternative genotype (n = 10) had significantly higher XOR activity compared to non-carriers (n = 19, *P* < 0.005). We confirmed this trend in the regression analysis adjusting for confounding factors (e.g., age and sex; *P*_*adjusted*_<0.01). Another two variants (rs206816 in promoter and rs206849 in intron 6) provided additional evidence for association with XOR activity showing a similar trend (*P*_*adjusted*_<0.039 and 0.011, respectively).

### An *XDH* variant is associated with higher risk of death in sepsis patients with ARDS

We tested whether *XDH* variants were associated with 60-day mortality. Coding missense SNP rs17011368 (T/C, Ile703Val) in exon 20 yielded marginal signal for association with increased mortality among 297 EA sepsis patients with ARDS (*P* < 0.043; OR = 2.87, 95% CI: 1.03–8.01). With each additional copy of the minor C allele at rs17011368, there was a 2.9-fold increase in mortality. Carriers of the Ile/Val or TC genotype had a mortality of 68.8% (11/16) at 60-days as compared to ARDS patients carrying the Ile/Ile or TT genotype (37%, 97/262). Kaplan-Meier survival analyses suggested that heterozygous (Ile/Val or TC) EA septic patients with ARDS had reduced survival rate compared to the homozygotes carrying Ile/Ile or TT (*P* = 0.038, Supplementary Fig. 3, Additional File 1). No association was observed in 158 AA sepsis patients with ARDS, partly due to a lack of statistical power in this smaller group.

### *XDH* SNPs are associated with sepsis-associated ARDS in a replication cohort of european descent

Replication was conducted on 590 sepsis patients of European descent (316 without and 274 with ARDS) enrolled as part of the GEN-SEP study [31]. Three intronic variants of *XDH* gene were nominally significant, including rs513311 (intron 3) (OR = 2.10, 95% CI = 1.14–3.85, *P* = 0.017; rs561525 (intron 9) (OR = 2.18, 95% CI = 1.04–4.59, *P* = 0.040), and rs2163059 (intron 12) (OR = 0.75, 95% CI 0.58–0.96, *P* = 0.026). Two of these variants were located in introns 9 and 12, a region harboring multiple variants for high risk of sepsis among AA patients in the CELEG cohort.

### Functional relevance of *XDH* variants

*In-silico* approaches were used to investigate potential biological consequences of identified lead variants associated with sepsis (Table 5 and Supplementary Results, Additional File 1). Noncoding risk loci are involved in the regulation of transcriptional activity and are enriched in eQTLs and cis-regulatory elements (CREs) [36]. *First*, two non-coding variants (rs513311 and rs2163059) displaying high regulatory potential (as suggested by RegulomeDB) were the lead variants associated with risk of sepsis, and were replicated in the GEN-SEP cohort. *Second*, three variants in the promoter and intronic regions (rs206816, rs206849 and rs185925) were associated with XOR activity, including rs185925 which provided the most compelling evidence for association with risk of sepsis. *Third*, SiNoPsis classified variants rs206849 (associated with XOR activity) and rs4952085 (associated with renal dysfunction) as creSNPs that could potentially be modifying CRE functions (e.g., disrupting histone marks and chromatin states). *Finally*, the missense variant rs17011368 that associated with mortality among sepsis patients with ARDS provided the highest Combined Annotation-Dependent Depletion (CADD) value of 22.4, supporting probable deleteriousness.

**Table 5:** Functional annotation of 28 *XDH* variants and their relevance for sepsis according to findings in this study

SNPs	RegulomedB rank (score)	eQTLs (GTEx) Tissue-specific p ≤ 0.05	SiNoPsis*	CADD (scaled)	Relevance for sepsis
rs206816	Other (7, 0.18412)	9 tissues (in cultured fibroblasts: p = 3.8e-7)		2.26	Associated with XOR activity among sepsis patients with ARDS
rs7575607	Other (7, 0.51392)	11 tissues (in cultured fibroblasts: p = 7.2e-6)		0.783	
rs206811	eQTL + TF binding/DNase peak (1f, 0.55436)	8 tissues (in cultured fibroblasts: p = 3.5e-6)		1.22	
rs206805	Other (7, 0.51392)	5 tissues		1.81	Associated with risk of sepsis
rs1366814	eQTL + TF binding/DNase peak (1f, 0.08217)	2 tissues		0.446	
rs513311	eQTL + TF binding/DNase peak (1f, 0.66703)			1.58	Associated with risk of sepsis & replicated in the GEN-SEP cohort
rs6714794	eQTL + TF binding/DNase peak (1f, 0.55436)	1 tissue		5.17	
rs206859	eQTL + TF binding/DNase peak (1f, 0.19549)	9 tissues (in cultured fibroblasts: p = 3.3e-6)		4.12	
rs206849	eQTL + TF binding/DNase peak (1f, 0.66703)	5 tissues	creSNP	0.841	Associated with XOR activity among sepsis patients with ARDS
rs13418515	Other (7, 0.51392)	2 tissues		4.05	
rs185925	eQTL + TF binding/DNase peak (1f, 0.55436)	7 tissues (in cultured fibroblasts: p = 1.3e-5)		6.84	Associated with risk of sepsis and XOR activity
rs561525	TF binding or DNase peak (5, 0.13454)			0.0560	Associated with risk of sepsis & replicated in the GEN-SEP cohort
rs17011403	TF binding + DNase peak (4, 0.60906)			0.192	
rs1864280	eQTL + TF binding + any motif + DNase footprint + DNase peak (1b, 0.98786)	3 tissues	creSNP	6.25	
rs2163059	eQTL + TF binding + any motif + DNase footprint + DNase peak (1b, 0.99571)	1 tissue		0.339	Associated with risk of sepsis & replicated in the GEN-SEP cohort
rs2281547	eQTL + TF binding/DNase peak (1f, 0.22271)	3 tissues		6.05	
rs1366817	Other (7, 0.51392)	3 tissues		5.68	
rs17011368	TF binding or DNase peak (5, 0.13454)			22.4	Associated with mortality among sepsis patients with ARDS
rs2281550	eQTL + TF binding/DNase peak (1f, 0.22271)	2 tissues		0.975	
rs13387204	eQTL + TF binding/DNase peak (1f, 0.08)	2 tissues		6.54	Associated with risk of sepsis
rs12621192	eQTL + TF binding/DNase peak (1f, 0.22271)	3 tissues		0.429	
rs1884725	eQTL + TF binding/DNase peak (1f, 0.02)	1 tissue		11.5	Associated with renal dysfunction among sepsis patients
rs4952085	eQTL + TF binding/DNase peak (1f, 0.55436)	1 tissue	creSNP	0.307	
rs207432	eQTL + TF binding/DNase peak (1f, 0.55436)	2 tissues		0.844	
rs207431	TF binding or DNase peak (5, 0.64)			0.0120	
rs932559	Motif hit (6, 0.0575)	1 tissue		2.52	
rs12478111	eQTL + TF binding/DNase peak (1f, 0.22271)	1 tissue		0.839	
rs6756897	eQTL + TF binding/DNase peak (1f, 0.55324)	2 tissues		1.19	

*Cis-regulatory elements (CREs) are the regions of non-coding DNA, which play a critical role in gene expression regulation. creSNPs are defined as variants that could potentially be modifying CRE regions/functions, according to the SNP classification scheme in SiNoPsis

## Discussion

This is the first study reporting an association between *XDH* polymorphisms and sepsis and sepsis-associated ARDS. The major findings of our study are: (i) *XDH* variants may increase the risk of sepsis and renal dysfunction as well as risk of death in sepsis-associated ARDS; (ii) XOR activity may serve as a biomarker for evaluating disease prognosis, and (iii) knowledge of *XDH* SNPs could potentially identify sub-groups that might benefit from development of targeted therapies to improve clinical outcomes.

Sepsis is a major cause of death worldwide [3, 37, 38] and is characterized by a dysregulated acute inflammatory response, frequently leading to multisystem organ failure. Oxidant injury related to formation of ROS (generated by XOR and other pro-oxidant enzymes) has been postulated in the pathogenesis of sepsis and organ dysfunction [39]. We found that XOR mediated damage leads to enhanced lung injury whereas inhibition of XOR with allopurinol is protective [16, 40, 41]. We also reported that XOR prevents restoration of the endothelial barrier (key to resolution of lung injury), and XOR inhibition improved survival in an endotoxemia murine model [42]. Despite a role of XOR in sepsis and ARDS, their utility as genetic markers for identifying patients at high probability of fatal outcome remains unclear.

The gene *XDH* encodes XOR, spans 80.42 kb on chromosome 2p23.1 and contains 36 exons. This gene has been tested for association with diseases including purine-pyrimidine metabolism and xanthinuria (type I), and may participate in pathways and processes including free radical induced apoptosis, purine metabolism, electron transport, lactation, and epithelial cell differentiation. *XDH* variants have been associated with xanthinuria [43], and recently with systemic hypertension and oxidative stress [44]. Activation of XOR may contribute to ARDS [41], and variation in XOR activity could be used to predict outcomes, i.e., XOR activity was increased in non-survivor septic patients [45].

Ethnic disparities in sepsis prevalence and outcome have been reported [6]. In the CELEG cohort, AA sepsis patients with ARDS had a higher mortality than in the ARDS Network trials [46]. These findings highlight a genetic predisposition among AA patients and warrant further study to explore a potential mechanistic basis for targeted therapy [19]. In our study, we observed association with risk of sepsis and renal dysfunction among AA patients. The most compelling signals derived from a cluster of three markers (rs185925, rs561525, rs2163059) from a region spanning introns 8 to 12. Indirect functional assessment provided further supporting evidence for potential functional consequences of these SNPs. First, we assessed patterns of LD, which is important in mapping genes associated with complex diseases. That region was in relatively strong LD among AA control subjects compared to the EAs (Supplementary Fig. 1, Additional File 1). Second, this region corresponds to amino acid position 245 to 404 (NP_000370), which aligns with “FAD_binding_4 super family (cl10516)”, a family with various enzymes that are similar to XOR and use FAD as a co-factor (Fig. 1, panel C). Third, this region was also enriched with regulatory elements identified by ENCODE regulatory tracks (Supplementary Fig. 2, Additional File 1). Finally, multiple association signals discovered for susceptibility of ARDS from the replication cohort of Spanish sepsis patients, highlighted the potential importance of this region influencing sepsis outcome. However, we cannot rule out the potential functional relevance of any of the markers studied in this region, as they maybe in LD with some causal variant(s) rather than being causal themselves. Additionally, two common SNPs in tight LD (including a coding-synonymous SNP rs1884725 in exon 27) provided the strongest evidence for association with risk of renal dysfunction among AA sepsis patients. Carriers had an increase of serum creatinine concentration compared to non-carriers. Interestingly, the SNP rs1884725 aligns with “Ald_Xan_dh_C2 superfamily (cl10595)” (Fig. 1, panel C), the largest functional domain for XOR. Given the potential importance of these regions in influencing susceptibility to and outcome of sepsis, further studies are warranted, such as extensive sequencing of this genomic region to unravel the likely causal variants (i.e., unknown low-frequency variants with potential biological roles), and mechanistic studies to understand the causal role of the risk variants identified. Nevertheless, our findings may help to understand health disparities in terms of higher morbidity and mortality observed in this vulnerable population.

Of note, a coding non-synonymous SNP (rs17011368, Ile703Val), which predicts a replacement of isoleucine by valine at amino acid position 703, was associated with 60-day mortality among EA sepsis patients with ARDS. This finding, coupled with a recent observation showing markedly enhanced activity of the Ile703Val variant [43], provides a compelling link between enhanced XOR activity and mortality in ARDS. Oxidative stress was identified as a key lung injury pathway controlling ARDS severity in animal models [47]. Recently reported associations between several oxidant/antioxidant pathway genes and ARDS highlight the importance of regulation of oxidant-mediated response in ARDS. It is likely that multiple genes in the same or interacting biological pathways contribute to sepsis/ARDS severity and outcomes. Thus, exploring the possible gene-gene interactions between *XDH* and other oxidative stress pathway genes (*e.g., NRF2*), or innate immunity genes (*e.g., CD14* and TLRs), will be important for future research.

For prioritization of sepsis-associated causal variants, especially those noncoding *XDH* variants, we applied multiple tools integrating various genomic and epigenomic annotations to elucidate multiple aspects of biological functionality of *XDH* variants. Multiple lines of evidence were discovered supporting our hypothesis that prioritized variants identified in our study could affect *XDH* expression and/or function, therefore influencing the severity and outcome of sepsis (Table 5). Further investigation to validate prioritized *XDH* variants on their functional impact in experimental studies is warranted.

We acknowledge some limitations of our study. Although our findings suggest that *XDH* variants are risk factors for sepsis, it deserves further validation in non-white cohorts with a larger sample size of sepsis cases. We found higher serum XOR activity in the combined group of sepsis patients, and sepsis patients with ARDS had the highest level of XOR. Further evidence was found for the lead variant rs185925, which was associated with XOR activity in a small subset of AA sepsis patients with ARDS (*P* < 0.005). These findings suggest that XOR activity is a genetically determined biomarker for sepsis and sepsis associated ARDS but clearly warrants further investigation. The high mortality rate in sepsis patients is likely due to environmental and genetic factors, which influence the host response to infection. However, we were unable to explore the genotype by environment (GxE) interactions in our study to confirm the possible modifying effects of local tissue/cell type specific environment (e.g., systemic inflammation) on *XDH* genetic risk factors in sepsis. With its capacity to generate ROS, XOR has been implicated in the development of tissue oxidative damage in a wide variety of respiratory and cardiovascular disorders such as ARDS, ischemia reperfusion injury, atherosclerosis, heart failure, and arterial hypertension [14, 48]. Other conditions where XOR has also been incriminated include COPD, obstructive sleep apnea, pulmonary manifestations of sickle cell disease, interstitial pneumonitis, as well as rejection of heart-lung transplant [14, 49]. Thus, our study may have a broader implication in assessing genetic influence of *XDH* on disease susceptibility and outcome wherever oxidative damage is involved.

## Conclusions

In summary, this is the first report to confirm that genetic markers in *XDH* are associated with sepsis and sepsis-associated ARDS in two diverse populations. The ethnic differences in risk alleles that we observed could explain some of the health disparity (i.e., higher morbidity and mortality) noted in AA patients. Although XOR activity was tested in a limited number of serum samples, our results confirm it may serve as a biomarker for assessing prognosis in these patients. Knowledge of *XDH* genetic polymorphisms could potentially identify sub-groups that might preferentially benefit from development of targeted therapies to improve the clinical outcomes of these devastating disease processes.

## Electronic supplementary material

Below is the link to the electronic supplementary material.

Additional file 1: Supplementary methods; supplementary results; references; supplementary tables 1 & 2; supplementary figures 1, 2 & 3

## Data Availability

The datasets used and/or analyzed during the current study are available from the corresponding author on reasonable request.

## Abbreviations

ARDS: Acute Respiratory Distress Syndrome
XOR: Xanthine Oxidoreductase
SNP: Single Nucleotide Polymorphisms
EA: European American
AA: African American
LD: Linkage Disequilibrium
ROS: Reactive Oxygen Species
HWE: Hardy-Weinberg Equilibrium
ENCODE: Encyclopedia of DNA Elements
