# Supplementary material for: Xanthine oxidoreductase gene polymorphisms are associated with high risk of sepsis and organ failure
Source: Respir Res. 2023 Jul 6;24:177. doi: 10.1186/s12931-023-02481-8 (PMC10324226; doi:10.1186/s12931-023-02481-8)

***Online Supplemental Digital Content***

Xanthine oxidoreductase gene polymorphisms are associated with high risk of sepsis and organ failure

Li Gao, MD, PhD^1,^*; Nicholas Rafaels, MS^2^; Tanda M. Dudenkov, PhD^3^; Mahendra Damarla, MD^1^; Rachel Damico, MD, PhD^1^; James P. Maloney, MD^4^; Marc Moss, MD^4^; Greg S. Martin, MD, MSc^5^; Jonathan Sevransky, MD, MHS^5^; Carl Shanholtz, MD^6^; Dan L. Herr, MD^6^; Joe G.N. Garcia, MD^7^; Tamara Hernandez-Beeftink, PhD^8,9^; Jesús Villar, MD, PhD^9,10,11^; Carlos Flores, PhD^8,10,12,13^; Terri H. Beaty, PhD^3^; Roy Brower, MD^1^; Paul M. Hassoun, MD^1,^*; Kathleen C. Barnes, PhD^2,^*

^1^Department of Medicine, The Johns Hopkins University School of Medicine, Baltimore, MD, USA

^2^Division of Biomedical Informatics & Personalized Medicine, University of Colorado School of Medicine, Aurora, CO, USA

^3^Department of Epidemiology, Bloomberg School of Public Health, The Johns Hopkins University, Baltimore, MD, USA

^4^Division of Pulmonary Sciences and Critical Care Medicine, University of Colorado School of Medicine, Aurora, CO, USA

^5^Department of Medicine, Emory University School of Medicine, Atlanta, GA, USA

^6^University of Maryland School of Medicine, Baltimore, MD, USA

^7^University of Arizona College of Medicine, Tucson, AZ, USA

^8^Research Unit, Hospital Universitario Ntra. Sra. de Candelaria, Santa Cruz de Tenerife, Spain

^9^Research Unit, Hospital Universitario Dr. Negrin, Las Palmas de Gran Canaria, Spain

^10^CIBER de Enfermedades Respiratorias (CIBERES), Instituto de Salud Carlos III, Madrid, Spain;

^11^Li Ka Shing Knowledge Institiute at St. Michael’s Hospital, Toronto, Canada

^12^Genomics Division, Instituto Tecnológico y de Energías Renovables, Santa Cruz de Tenerife, Spain

^13^Facultad de Ciencias de la Salud, Universidad Fernando Pessoa Canarias, Las Palmas de Gran Canaria, Spain

**Supplementary MethodS**

**Study Subjects**

***CELEG cohort (discovery dataset)***

The Consortium to Evaluate Lung Edema Genetics (CELEG) study enrolled 974 patients (EA=621, AA=353) with sepsis or septic shock. The European American dataset had 621 subjects, including: (i) patients with sepsis-associated acute respiratory distress syndrome (ARDS) (n=297), and (ii) patients with sepsis alone without ARDS (n=324). An independent African American dataset had 353 subjects (158 with ARDS and 195 with sepsis). Definition of sepsis met the criteria of the Third International Consensus Definitions for Sepsis and Septic Shock (Sepsis-3) (1), all CELEG patients with sepsis also met prior definitions for severe sepsis (2). Admission to intensive care units (ICUs) was a requirement for enrollment (subjects were enrolled within 72 hours of admission in participating intensive care units), and all patients had sepsis or septic shock. All ARDS patients had a PaO_2_/FiO_2_ ratio <300 mmHg, with sepsis as the predisposing illness (patients with a non-sepsis cause of ARDS were not enrolled). Exclusion criteria were allogeneic bone marrow transplant and severe leukopenia (WBC<1000/ul). APACHE II scores (3) were recorded to ensure comparability of severity of illness between sepsis and ARDS groups, we used the most abnormal values documented during the 24-hour period following whichever sepsis and or ARDS qualifying criteria were met. Controls (EA=302, AA=406) were defined as subjects without any recent acute illness or any chronic illness requiring medical care.

***GEN-SEP cohort (replication dataset)***

Sepsis was defined according to the Third International Consensus Definitions for Sepsis (1). Patients were admitted into a network of ICUs in Spain (GEN-SEP). This cohort was previously used to conduct a sepsis-associated ARDS GWAS on 590 unrelated patients (274 sepsis-associated ARDS cases and 316 controls with sepsis only) (4).

**Statistical Analyses**

***Ancestry/Admixture Estimation***

To estimate African and European ancestry for the African American subjects, 40 ancestry informative markers (AIMs) were selected with large differences in allele frequencies between “continental” ancestral Yoruba Nigerians and CEPH populations in the HAPMAP database ([www.hapmap.org](http://www.hapmap.org)) (Table E2). All SNPs were in Hardy-Weinberg equilibrium (p>10^-6^) among ARDS cases, sepsis cases, and controls, and had inter-marker distances greater than 5 Mb. We used STRUCTURE (5) admixture modeling to determine individual and subgroup ancestry probabilities using HapMap Yoruba Nigerians (YRI) and CEU samples to approximate the parental populations. An *a priori* number of clusters were specified to be k=2, allele frequencies were assumed to be independent, and we ran 10,000 burnins and 10,000 MCMC iterations. The non-parametric Wilcoxon rank sum test was used to test for differences in distribution of ancestry proportions among the three groups.

***Genetic association testing in the GEN-SEP cohort***

SNP data were obtained for 587,352 sites using the Axiom Genome-Wide Human CEU 1 Array (Affymetrix, Santa Clara, CA, USA). SNPs were imputed based on the HRC release 1.1.0, and variants with low allele frequency [minor allele frequency (MAF) <1%] or with a low imputation quality (Rsq<0.3) were excluded from the analysis. The methods for the GWAS of ARDS or sepsis have been described elsewhere (4).

**Supplementary RESULTS**

***Variant*** ***Frequencies, Linkage Disequilibrium and Population Structure***

The 28 *XDH* SNPs spanning 95Kb on chromosome 2 are listed in **Supplementary Table 1**. All SNPs were in HWE, and three SNPs had MAF <5% among European Americans. Patterns of LD were similar across the two ethnic groups (**Supplementary Figure 1**). Notably, strong LD was observed in a region encompassing exons 8 to 13 in the African Americans, whereas LD was low in European Americans since two markers (rs561525 and rs17011403) were rare or monomorphic in European Americans. Population structure analyses in the African Americans group revealed no significant differences in the average European ancestry (admixture) of 21.7% among sepsis cases and 22.1% among the controls (**Supplementary Table 2**).

***Functional relevance of XDH variants***

*In-silico* approaches were used to investigate potential biological consequences of identified lead variants associated with sepsis (**Table 5**). *First*, predicted functional effect (regulatory potential) for variants in non-coding or intergenic regions was ranked by RegulomeDB (between 1 and 7, with 1 being the highest scoring which are likely to affect both transcription factor binding and expression of XDH). The variant rs2163059 (intron 12) yielded highest scores, along with another two variants (rs513311 in intron 3 and rs1864280 in intron 11). Interestingly, two out of three variants (rs513311 and rs2163059) displaying high regulatory potential were the lead variants identified in this study associated with risk of sepsis, and also replicated in the GEN-SEP cohort. *Second*, we explored the GTEx database and discovered further evidence on significant eQTLs in more than 5 tissue types including the cultured fibroblasts for 7 variants (rs206816 [promoter], rs7575607 [promoter], rs206811 and rs206805 [intron 1], rs206859 and rs206849 [intron 6] as well as rs185925 [intron 8]). Indeed, three of these (rs206816, rs206849 and rs185925) were associated with XOR activity among AA sepsis patients with ARDS, including rs185925 which provided the most compelling evidence for association with risk of sepsis. *Third*, we explored CREs utilizing SiNoPsis. CREs include promoters and enhancers as well as noncoding sequences, either near to or far from genes, which include binding sites for the regulatory factors required for the expression of the gene. SiNoPsis classifies three variants (rs206849 [intron 6], rs1864280 [intron 11] and rs4952085 [intron 27]) as creSNPs that could potentially be modifying CRE functions (e.g., disrupting histone marks and chromatin states). Of note, rs206849 was associated with XOR activity whereas rs4952085 was associated with renal dysfunction among AA sepsis patients. *Finally*, using Open Targets Genetics, we generated scaled Combined Annotation-Dependent Depletion (CADD), which is a widely used measure of variant deleteriousness that can effectively prioritize causal variants in genetic analyses, supporting probable deleteriousness when >20 (*i.e.*, among the variants in over the 1% of most deleterious). The missense variant rs17011368 that associated with mortality among sepsis patients with ARDS provided the highest value of 22.4, among all 28 variants assessed.

**REFERENCES**:

1. Singer M, Deutschman CS, Seymour CW, Shankar-Hari M, Annane D, Bauer M, Bellomo R, Bernard GR, Chiche JD, Coopersmith CM, Hotchkiss RS, Levy MM, Marshall JC, Martin GS, Opal SM, Rubenfeld GD, van der Poll T, Vincent JL, Angus DC. The Third International Consensus Definitions for Sepsis and Septic Shock (Sepsis-3). *Jama* 2016; 315: 801-810.

2. Levy MM, Fink MP, Marshall JC, Abraham E, Angus D, Cook D, Cohen J, Opal SM, Vincent JL, Ramsay G, Sccm/Esicm/Accp/Ats/Sis. 2001 SCCM/ESICM/ACCP/ATS/SIS International Sepsis Definitions Conference. *Crit Care Med* 2003; 31: 1250-1256.

3. Knaus WA, Draper EA, Wagner DP, Zimmerman JE. APACHE II: a severity of disease classification system. *Crit Care Med* 1985; 13: 818-829.

4. Guillen-Guio B, Lorenzo-Salazar JM, Ma SF, Hou PC, Hernandez-Beeftink T, Corrales A, Garcia-Laorden MI, Jou J, Espinosa E, Muriel A, Dominguez D, Lorente L, Martin MM, Rodriguez-Gallego C, Sole-Violan J, Ambros A, Carriedo D, Blanco J, Anon JM, Reilly JP, Jones TK, Ittner CA, Feng R, Schoneweck F, Kiehntopf M, Noth I, Scholz M, Brunkhorst FM, Scherag A, Meyer NJ, Villar J, Flores C. Sepsis-associated acute respiratory distress syndrome in individuals of European ancestry: a genome-wide association study. *The Lancet Respiratory medicine* 2020; 8: 258-266.

5. Pritchard JK, Stephens M, Donnelly P. Inference of population structure using multilocus genotype data. *Genetics* 2000; 155: 945-959.

**Supplementary Table 1. Chromosome 2 location, inter-SNP distance, alleles, type and minor allele frequency (MAF) of 28 single nucleotide polymorphisms in gene *XDH.***

| **Marker** | **Chr.2 Position**  **(hg19)** | **Alleles** | **Inter-SNP distance (bp)** | **Type/Relative position (amino acid change)** | **MAF in EA (N)** | **MAF in AA (N)** |
| --- | --- | --- | --- | --- | --- | --- |
| rs206816 | 31644983 | G/A | 0 | Promoter, -7451 | 0.3969 (262) | 0.3077 (403) |
| rs7575607 | 31640455 | A/G | 4528 | Promoter, -2923 | 0.3853 (279) | 0.3872 (399) |
| rs206811 | 31636915 | C/T | 954 | Intron 1, 617 | 0.3676 (272) | 0.2827 (405) |
| rs206805 | 31630867 | A/G | 6048 | Intron 1, 6665 | 0.2015 (278) | 0.2741 (406) |
| rs1366814 | 31628705 | A/C | 2162 | Intron (boundary), 2+68 | 0.1093 (279) | 0.1207 (406) |
| rs513311 | 31625610 | C/A | 3095 | Intron 3, 11922 | 0.0343 (277) | 0.125 (404) |
| rs6714794 | 31623810 | T/C | 1800 | Intron 4, 13722 | 0.04301 (278) | 0.09901 (404) |
| rs206859 | 31619559 | C/T | 4251 | Intron 6, 17973 | 0.3495 (279) | 0.3286 (392) |
| rs206849 | 31613249 | C/T | 6310 | Intron 6, 24283 | 0.3907 (279) | 0.2231 (399) |
| rs13418515 | 31612781 | C/T | 468 | Intron 6, 24751 | 0.1475 (278) | 0.163 (405) |
| rs185925 | 31609993 | T/C | 2788 | Intron 8, 27539 | 0.3315 (279) | 0.2298 (396) |
| rs561525 | 31608855 | T/C | 1138 | Intron 9, 28677 | 0.0179 (279) | 0.0988 (405) |
| rs17011403 | 31608435 | C/T | 420 | Intron 9, 29097 | 0 (279) | 0.0727 (406) |
| rs1864280 | 31605615 | A/G | 2820 | Intron 11, 31917 | 0.4534 (279) | 0.3094 (404) |
| rs2163059 | 31603134 | A/G* | 2481 | Intron 12, 34398 | 0.3459 (279) | 0.4117 (402) |
| rs2281547 | 31598823 | C/T | 3968 | Intron 14, 38709 | 0.3849 (278) | 0.3049 (405) |
| rs1366817 | 31592403 | A/G | 6420 | Intron 18, 45129 | 0.3651(278) | 0.3837 (404) |
| rs17011368 | 31590917 | T/C | 1486 | Coding exon (20), 46615 (I703V) | 0.0475 (263) | 0.1277 (42) |
| rs2281550 | 31590759 | C/T | 158 | Intron (boundary), 20+68 | 0.3495 (279) | 0.2463 (406) |
| rs13387204 | 31589654 | C/T | 1105 | Intron (boundary), 21+82 | 0.3548 (279) | 0.3545 (402) |
| rs12621192 | 31588664 | C/T | 990 | Intron, 48868 | 0.2921 (279) | 0.0831 (403) |
| rs1884725 | 31571786 | G/A | 16878 | Coding exon (27), 65746 (F1010F) | 0.2266 (278) | 0.201 (403) |
| rs4952085 | 31570689 | A/G | 1097 | Intron, 66843 | 0.2294 (279) | 0.2035 (403) |
| rs207432 | 31553987 | A/C | 9810 | Downstream, 83545 | 0.3845 (277) | 0.4261 (406) |
| rs207431 | 31553394 | A/G | 593 | Downstream, 84138 | 0.1685 (279) | 0.3148 (405) |
| rs932559 | 31552902 | C/A | 492 | Downstream, 84630 | 0.4442 (276) | 0.1638 (406) |
| rs12478111 | 31550180 | G/A | 2722 | Downstream, 87352 | 0.2258 (279) | 0.1675 (406) |
| rs6756897 | 31549757 | T/C | 423 | Downstream, 87775 | 0.3022 (278) | 0.3864 (405) |

**Supplementary Table 2.** **List of 40 ancestry informative markers with minor allele frequencies^a^**

| **MARKER** | **CHROM** | **POSTION** | **ARDS** | |  | **Sepsis** | |  | **Controls** | |
| --- | --- | --- | --- | --- | --- | --- | --- | --- | --- | --- |
|  |  |  | **MAF** | **HWE-p** |  | **MAF** | **HWE-p** |  | **MAF** | **HWE-p** |
| rs6003 | 1 | 195297644 | 0.37 | 0.433 |  | 0.39 | 0.8745 |  | 0.41 | 0.918 |
| rs630101 | 1 | 234839039 | 0.17 | 0.750 |  | 0.15 | 0.0726 |  | 0.19 | 0.654 |
| rs2642995 | 1 | 245239188 | 0.33 | 0.415 |  | 0.33 | 0.0103 |  | 0.34 | 0.503 |
| rs880143 | 1 | 245934736 | 0.16 | 0.037 |  | 0.20 | 0.8144 |  | 0.18 | 0.004 |
| rs2339475 | 2 | 29853294 | 0.19 | 0.549 |  | 0.18 | 0.7960 |  | 0.18 | 1.000 |
| rs2625051 | 2 | 131229320 | 0.17 | 0.340 |  | 0.21 | 0.0176 |  | 0.20 | 0.757 |
| rs4675966 | 2 | 241893015 | 0.33 | 0.678 |  | 0.26 | 0.7005 |  | 0.32 | 0.491 |
| rs7611703 | 3 | 2749996 | 0.21 | 0.580 |  | 0.20 | 0.8144 |  | 0.21 | 1.000 |
| rs4596126 | 3 | 13634898 | 0.26 | 0.050 |  | 0.18 | 0.6049 |  | 0.19 | 0.076 |
| rs2686085 | 3 | 198707095 | 0.42 | 0.023 |  | 0.50 | 0.5463 |  | 0.48 | 0.765 |
| rs758973 | 4 | 13148471 | 0.25 | 0.627 |  | 0.30 | 1.0000 |  | 0.25 | 0.034 |
| rs1525760 | 4 | 117354829 | 0.19 | 0.379 |  | 0.22 | 0.1880 |  | 0.23 | 0.332 |
| rs1352695 | 4 | 158781719 | 0.21 | 0.164 |  | 0.18 | 0.4520 |  | 0.18 | 0.870 |
| rs930072 | 5 | 36701828 | 0.19 | 0.770 |  | 0.20 | 0.0948 |  | 0.18 | 0.405 |
| rs874973 | 5 | 72773651 | 0.28 | 0.116 |  | 0.26 | 0.6916 |  | 0.29 | 0.022 |
| rs3317 | 5 | 112240050 | 0.16 | 0.734 |  | 0.13 | 0.7428 |  | 0.14 | 0.398 |
| rs185493 | 5 | 177923864 | 0.10 | 1.000 |  | 0.12 | 0.7113 |  | 0.12 | 0.232 |
| rs727619 | 6 | 170548119 | 0.36 | 1.000 |  | 0.44 | 0.3526 |  | 0.39 | 0.337 |
| rs1011024 | 7 | 99035337 | 0.19 | 0.539 |  | 0.18 | 0.1890 |  | 0.19 | 0.873 |
| rs963314 | 7 | 147493565 | 0.12 | 0.683 |  | 0.12 | 0.1471 |  | 0.10 | 0.785 |
| rs2045638 | 8 | 2963312 | 0.21 | 0.782 |  | 0.20 | 0.0145 |  | 0.20 | 0.217 |
| rs983271 | 8 | 35534194 | 0.45 | 0.572 |  | 0.43 | 0.5319 |  | 0.47 | 0.133 |
| rs3750203 | 8 | 144803169 | 0.18 | 1.000 |  | 0.12 | 0.2514 |  | 0.13 | 0.661 |
| rs3780293 | 9 | 79266067 | 0.16 | 0.497 |  | 0.13 | 0.4854 |  | 0.14 | 0.674 |
| rs7860423 | 9 | 140075368 | 0.30 | 1.000 |  | 0.23 | 0.0873 |  | 0.23 | 0.574 |
| rs2207782 | 10 | 83747179 | 0.20 | 0.568 |  | 0.26 | 0.1644 |  | 0.21 | 1.000 |
| rs1050755 | 10 | 112043589 | 0.17 | 0.340 |  | 0.18 | 0.7960 |  | 0.17 | 0.477 |
| rs905552 | 11 | 19052735 | 0.15 | 0.733 |  | 0.14 | 0.0004 |  | 0.14 | 0.288 |
| rs1042602 | 11 | 88551344 | 0.07 | 1.000 |  | 0.07 | 0.2376 |  | 0.07 | 0.137 |
| rs236919 | 11 | 116600571 | 0.19 | 1.000 |  | 0.22 | 0.2595 |  | 0.20 | 0.641 |
| rs959354 | 11 | 129514791 | 0.35 | 0.542 |  | 0.28 | 0.7079 |  | 0.32 | 0.257 |
| rs895898 | 13 | 38125547 | 0.30 | 0.826 |  | 0.26 | 1.0000 |  | 0.25 | 0.695 |
| rs3825663 | 14 | 89499502 | 0.29 | 1.000 |  | 0.24 | 0.5276 |  | 0.28 | 0.386 |
| rs188324 | 15 | 93501509 | 0.11 | 1.000 |  | 0.13 | 0.7428 |  | 0.15 | 0.845 |
| rs2891 | 17 | 3652275 | 0.11 | 0.628 |  | 0.09 | 0.6523 |  | 0.14 | 1.000 |
| rs1478785 | 17 | 70734460 | 0.18 | 0.214 |  | 0.16 | 0.0445 |  | 0.16 | 0.577 |
| rs4436849 | 18 | 28809444 | 0.42 | 0.088 |  | 0.36 | 0.5115 |  | 0.38 | 0.597 |
| rs6013031 | 20 | 48907998 | 0.11 | 0.117 |  | 0.08 | 0.0026 |  | 0.11 | 1.000 |
| rs2064056 | 21 | 35516978 | 0.18 | 0.536 |  | 0.18 | 0.1196 |  | 0.15 | 0.230 |
| rs4821667 | 22 | 36160027 | 0.16 | 1.000 |  | 0.18 | 0.6129 |  | 0.20 | 0.027 |

**^a^** There was no significant evidence for racial admixture compared to self-reported race.

**Supplementary FigureS**

**Supplementary Figure 1.** The linkage disequilibrium (LD) structure (defined by pairwise r^2^) between SNPs in 406 African American (left panel) and 302 European American control subjects using the Haploview software. The exon/intron locations and relative positions of SNPs within gene *XDH* are also displayed in the middle panel.


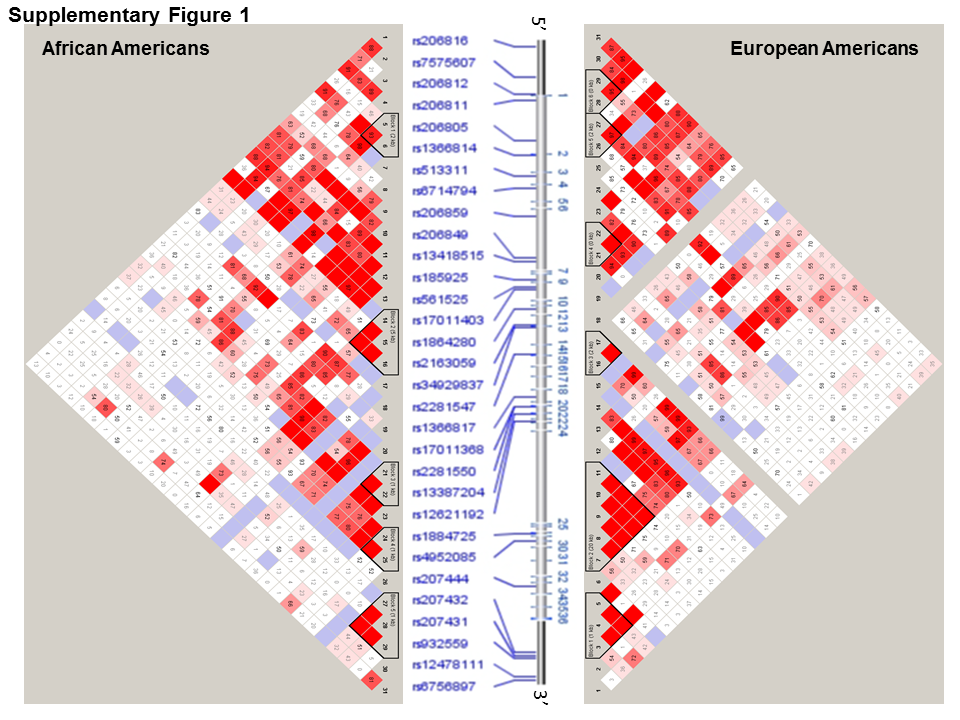


**Supplementary Figure 2.** This figure illustrates the *XDH* structure, position of twenty-seven SNPs within *XDH* gene region (User Track) and regulatory regions with ENCODE regulation tracks.


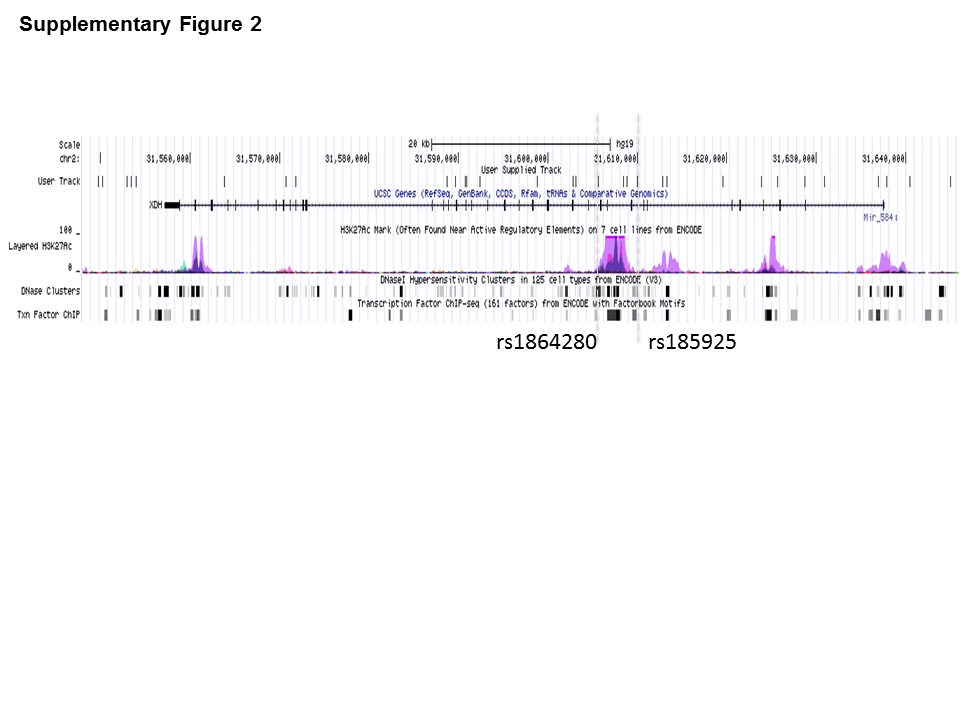


**Supplementary Figure 3.** Kaplan-Meier survival estimates of 60-day survival in European American sepsis patients with ARDS dependent on genotypes of rs17011368 (T/C, I703V) in *XDH*.


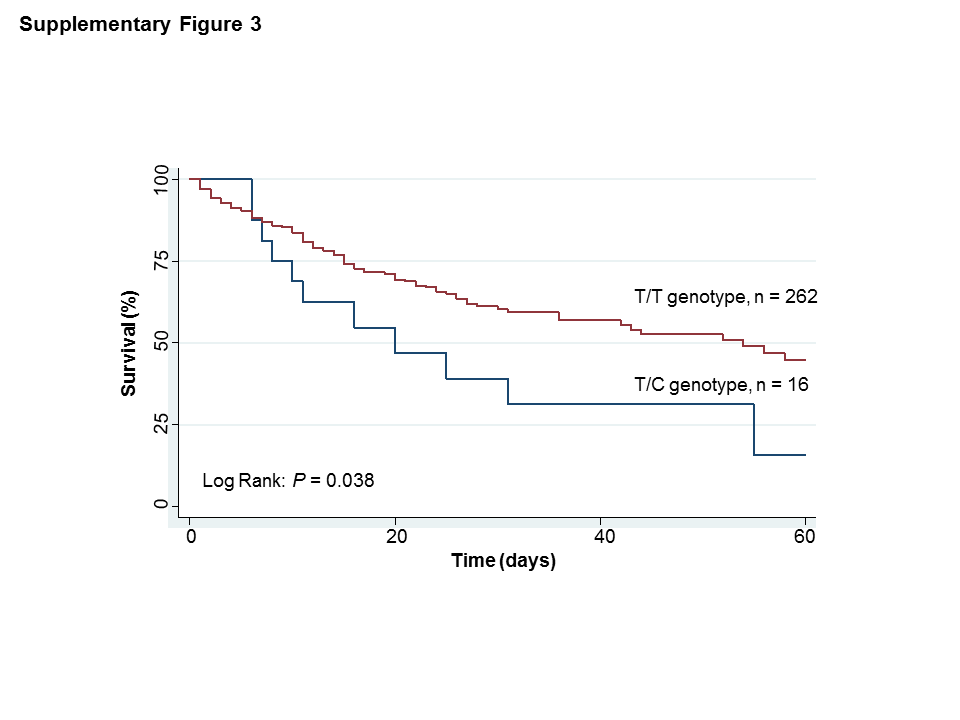

Supplement: Supplementary file 1 — Additional file 1: Supplementary methods; supplementary results; references; supplementary tables 1 & 2; supplementary figures 1, 2 & 3 [file 12931_2023_2481_MOESM1_ESM.docx]
